# Supplementary material for: Simultaneous Detection of Different Zika Virus Lineages via Molecular Computation in a Point-of-Care Assay
Source: Viruses. 2018 Dec 14;10(12):714. doi: 10.3390/v10120714 (PMC6316447; doi:10.3390/v10120714)
Supplement: Supplementary file 1 [file viruses-10-00714-s001.zip › viruses-383527-SI.pdf]

Simultaneous detection of different Zika virus lineages via molecular computation in a point-of-care assay

Sanchita Bhadra[1*], Miguel A. Saldaña[2], Hannah Grace Han[1], Grant L. Hughes[3,4], and Andrew D. Ellington[1]

Supplementary Table S1. Primers used for single and multiplex Zika LAMP assays.

NS5	NS5.BIP*	ATGCVCARATGTGGCAGCTYCTTCCCHGTTGGNACCCA
	NS5.B3	GTCATCCARTCTCCRTTRCC
	NS5.F3	ATCCATTGTGGTYCCYTGY
	NS5.FIP	GCHAGRCARGCAGTCTCMCGRGATGAAYTGATTGGCCGRGC
	NS5.LR	TGCTCCATCCYGCCCCYGGHGA
NS1	NS1.F3	GGGCCCAYCTGATYGAGAT
	NS1.B3	TCCACATGTYTCCTCCACR
	NS1.FIP	GTGGMCCAGCTAAAGACTTGGGAATGGCCAAAGTCYCACAC
	NS1.BIP	ACAGRACYCAAATGAAAGGGCCGGGCATTCCTCAAAYCGRAT
	NS1.LF	ATGGCACAGTGARGAGCTYGAA
Capsid	Capsid.F3	TGCTAAAACGCGGAGTAGC
	Capsid.B3	TGGCAGCCAGRTCYTTCTTR
	Capsid.FIP	ACCATYCTRATGGGCCCATGCCCTTTGGRGGYTTGAAG
	Capsid.BIP	TCACGGCAATCAARCCATCACTAGCCTCTTTYTTCCCCACKG
	Capsid.LF	GGGTCTCATCAAYAGATGGGGTW
	Capsid.SF	CTTGGCAATTCTAGCCTTYTTGAGAT
NS3	NS3.F3	ACGAAGACCAYGCACAYTG
	NS3.B3	ATTCCGGCAGATGCWACY
	NS3.FIP	TCGGCCTCAGGYCGATAGAGCAGCAAGAATGCTYCTTGACA
	NS3.BIP	AGCHGCCATTGAGGGAGAGTTCTAGGCCARCCARACRGGA
	NS3.LR	GAGGCTATGAGGCCATCYTGGAG

* Mixed base identity: **V** = A,C,G; **R** = A,G; **Y** = C,T; **H** = A,C,T; **N** = A,T,C,G; **M** = A,C; **K** = G,T; **W** = A,T

Supplementary Table S2. Strand exchange probes used in this study.

Capsid OSD	Capsid.FAM	RCCCAGCAGRAGTCCGGCYGGCARCCT/56-FAM/
	Capsid.Q	/5IABkFQ/AGGYTGCCRGCCGGACT/3InvdT/
NS5 OSD	NS5.Q	RAGGTCYCTTCTRTGGAAGTC/3IABkFQ/
	NS5.FAM	/56-FAM/GACTTCCAYAGAAGRGACCTYCGACTGATGGC/3InvdT/
NS1 OSD	NS1.FAM	/56-FAM/CATGATMAGATCACTYTCTTCYAYTCCATCTGTCCACAAT/3InvdT/
	NS1.Q	GGARTRGAAGARAGTGATCTKATCATG/3IABkFQ/
NS3 OSD	NS3.FAM	GAGCAAAGGAAGACCTTYGTGGAACTCATGAARAGAGGAGA/36-FAM/
	NS3.Q	/5IABkFQ/TCTCCTCTYTTCATGAGTTCCACRAAGGT/3InvdT/
CAN3 2GO probe	CAN3.2GO.FAM	RCCCAGCAGRAGTCCGGCYGGCARCCTGATCC/36-FAM/
	CAN3.2GO.Gate	GAGCAAAGGAAGACCTTYGTGGAACTCATGAARAGAGGAGAGGATCAGGYTGC CRGCCGGA /3InvdT/
	CAN3.2GO.Q	/5IABkFQ/TCTCCTCTYTTCATGAGTTCCACRAAGGT/3InvdT/
N5N1 2GO probe	N5N1.2GO.FAM	/56-FAM/CATGATMAGATCACTYTCTTCYAYTCCATCTGTCCACAAT/3InvdT/
	N5N1.2GO.Gate	GGARTRGAAGARAGTGATCTKATCATGGACTTCCAYAGAAGRGACCTYCGACT GATGGC/3InvdT/
	N5N1.2GO.Q	RAGGTCYCTTCTRTGGAAGTC/3IABkFQ/
4GO probe	4GO.S1	GTCYCTTCTRTGGAAGTCCTCCATGATMAGATCACTYTCTTCYAYTCCATCTGTC CACAAT/3InvdT/
	4GO.S2	/56-FAM/CAGGGAGGAGGACTTCCAYAGAAGRGACCTYCGACTGATGGC/3InvdT/
	4GO.S3	CAGCAGRAGTCCGGCYGGCARCCTGAGGAGGAGGGAG/3IABkFQ/
	4GO.S4	GAGCAAAGGAAGACCTTYGTGGAACTCATGAARAGAGGAGACTCCTCAGGYTG CCRGCCGG/3InvdT/
	4GO.S5	RAGTGATCTKATCATGTCCCTGCTCCCTTCTCCTCTYTTCATG/3InvdT/

Supplementary Table S3. LAMP synthetic template RNA sequences.

Zika NS3 RNA template	GGGAGGCAGGAAUCCCAACAAACCUGGAGAUGAGUAUCUGUAUGGAGGUGGGUGCGCAGAGAC<br>UGACGAAGACCAUGCACACUGGCUUGAAGCAAGAAUGCUCCUUGACAAUAUUUACCUCCAAGAU<br>GGCCUCAUAGCCUCGCUCUAUCGACCUGAGGCCGACAAAGUAGCAGCCAUUGAGGGAGAGUUCA<br>AGCUUAGGACGGAGCAAAGGAAGACCUUUGUGGAACUCAUGAAAAGAGGAGAUCUUCCUGUUU<br>GGCUGGCCUAUCAGGUUGCAUCUGCCGGAAUAACCUACACAGAUAGAAGAUGGUGCUUUGAUG<br>GCACGACCAACAACACCAUAAUGGAAGACAGUGUGCGAAUUCUGCAGAUAUCCAUCACACUGGCG<br>G
Zika NS1 RNA template	GGGCUGAAGAGGGCCCAUCUGAUCGAGAUGAAAACAUGUGAAUGGCCAAAGUCCCACACAUUGU<br>GGACAGAUGGAAUAGAAGAGAGUGAUCUGAUCAUACCCAAGUCUUUAGCUGGGCCACUCAGCCA<br>UCACAAUACCAGAGAGGGCUACAGGACCCAAAUGAAAGGGCCAUGGCACAGUGAAGAGCUUGAA<br>AUUCGGUUUGAGGAAUGCCCAGGCACUAAGGUCCACGUGGAGGAAACAUGUGGAACAAGAGGAC<br>CAUCUCUGAGAUCAACCACUGCAAGCGGAAGGGUGAUCGAGGAAUGGUGCUGCAGGGAGUGCAC<br>AAUGCCCCCACUGUCGUUCCGGGCUAAAGAUGGCUGUUGGUAUGGAAUGGAGAUAAGGCCCAG<br>GAAAGAACCAGAAAGCAACUUAGUAAGGUCAAUGGUGACUGCAGGAUCAAGAAUUCUGCAGAUA<br>UCCAUCACACUGGCGG
Zika Capsid RNA template	GGGCGAGAGUUUCUGGUCAUGAAAAACCCAAAAAAGAAAUCCGGAGGAUUCCGGAUUGUCAAUA<br>UGCUAAAACGCGGAGUAGCCCGUGUGAGCCCCUUUGGGGGCUUGAAGAGGCUGCCAGCCGGACU<br>UCUGCUGGGUCAUGGGCCCAUCAGGAUGGUCUUGGCAAUUCUAGCCUUUUUGAGAUUCACGGC<br>AAUCAAGCCAUCACUGGGUCUCAUCAAUAGAUGGGGUUCAGUGGGGAAAAAAGAGGCUAUGGA<br>AAUAAUAAAGAAGUUCAAGAAAGAUCUGGCUGCCAUGCUGAGAAUAAUCAAUGCUAGGAAGGA<br>GAAGAAGAAUUCUGCAGAUAUCCAUCACACUGGCGG
Zika NS5 RNA template	GGUAGAUCCAUUGUGGUCCCUUGCCGCCACCAAGAUGAAUUGAUUGGCCGAGCCCGUGUAUCAC<br>CAGGGGCAGGAUGGAGCAUUCGGGAGACUGCCUGUCUAGCAAAAUCAUAUGCACAGAUGUGGC<br>AGCUUCUUUACUUCCACAGAAGAGACCUUCGACUGAUGGCCAAUGCUAUUUGUUCGGCUGUGCC<br>AGUUGACUGGGUACCAACCGGGAGAACCACCUGGUCAAUCCACGGAAAGGGAGAAUGGAUGACU<br>ACUGAGGACAUGCUCAUGGUGUGGAAUAGAGUGUGGAUUGAGGAGGAAUUCUGCAGAUAUCCA<br>UCACACUGGCGGCCGCUCGAGC

Supplementary Table S4. Zika NS2b qRT-PCR primers, TaqMan probe, and RNA template.

Zika4481_F	CTGTGGCATGAACCCAATAG
Zika4552c_R	ATCCCATAGAGCACCACTCC
Zika4507cTqMFAM	/56-FAM/CCACGCTCCAGCTGCAAAGG/3IABkFQ/
NS2b qRT-PCR RNA template	GGGAC CAUCUGUGGCAUGAACCCAA UAGCCAUACC CUUUGCAGCU GGAGCGUGGU ACGUGUAUGU GAAGACUGGAAAAAGGAGUG GUGCUCUAUG GGAUGUGCCU

Supplementary Table S5. Viral genomic RNA samples used in this study.

BEI Resources catalog number	GenBank Accession number	Description of material
NR-50369	KX087101	Zika virus -- Zika virus, PRVABC59, Heat-Inactivated
NR-50085	AY632535	Genomic RNA from Zika Virus, MR 766 -- Zika virus
NR-50086	KU963574	Genomic RNA from Zika Virus, IbH 30656 -- Zika virus
NR-50241	KU820897	Genomic RNA from Zika Virus, FLR -- Zika virus
NR-50283	KX446950	Genomic RNA from Zika Virus, MEX 2-81 -- Zika virus
NR-50284	KX446951	Genomic RNA from Zika Virus, MEX I-7 -- Zika virus
NR-50244	KX087101	Genomic RNA from Zika Virus, PRVABC59 -- Zika virus
NR-50358	KX262887	Genomic RNA from Zika Virus, R103451 -- Zika virus
NR-50328	KX198135	Genomic RNA from Zika Virus, H/PAN/2016/-259634 -- Zika virus
NR-50329	KX156774	Genomic RNA from Zika Virus, H/PAN/2015/CDC-259359 -- Zika virus
NR-50330	KX156775	Genomic RNA from Zika Virus, H/PAN/2015/CDC-259249 -- Zika virus
NR-50331	KX156776	Genomic RNA from Zika Virus, H/PAN/2015/CDC-259364 -- Zika virus
NR-50345	AF192908.1	Genomic RNA from Chikungunya Virus, 181/25 -- Chikungunya virus
NR-32847	EU848545	Dengue virus -- Dengue Virus Nucleic Acid Panel: Dengue Virus Type 1, Hawaii, Dengue Virus Type 2, New Guinea C, Dengue Virus Type 3, Philippines/H87/1956, Dengue Virus Type 4, H241

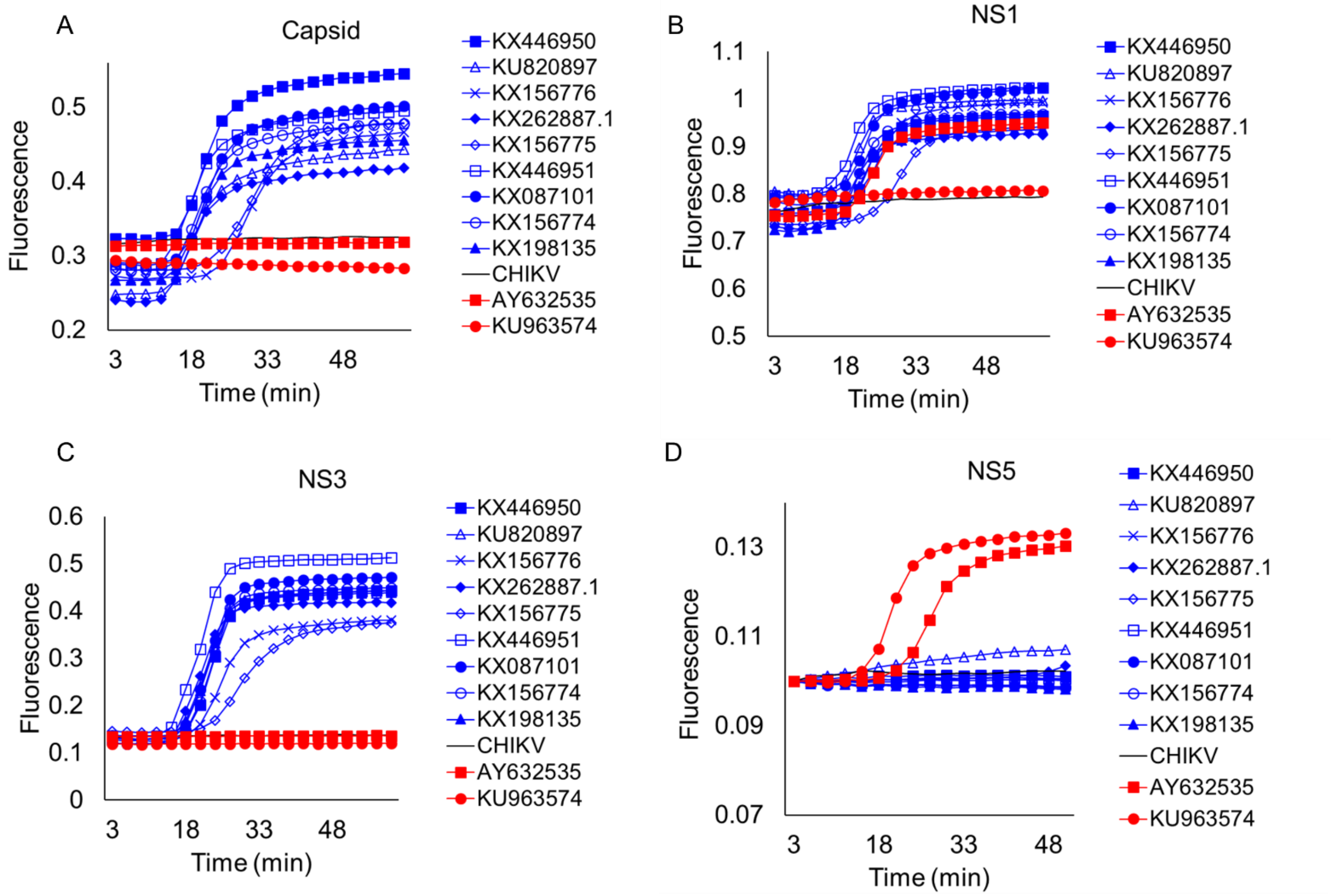

Supplementary Figure 1. Detection of Zika virus genomic RNA using non-degenerate primers in LAMP-OSD assays. Genomic RNA from CHIKV or Asian or African lineage ZIKV (indicated by their GenBank accession numbers) were used as templates for amplification assays for Zika virus capsid (panel A), NS1 (panel B), NS3 (panel C), and NS5 (panel D) genes. OSD fluorescence signals measured in real-time using LightCycler 96 real-time PCR machine are depicted as blue (Asian ZIKV), red (African ZIKV), and black (CHIKV) traces. Representative results from three replicate experiments are depicted.

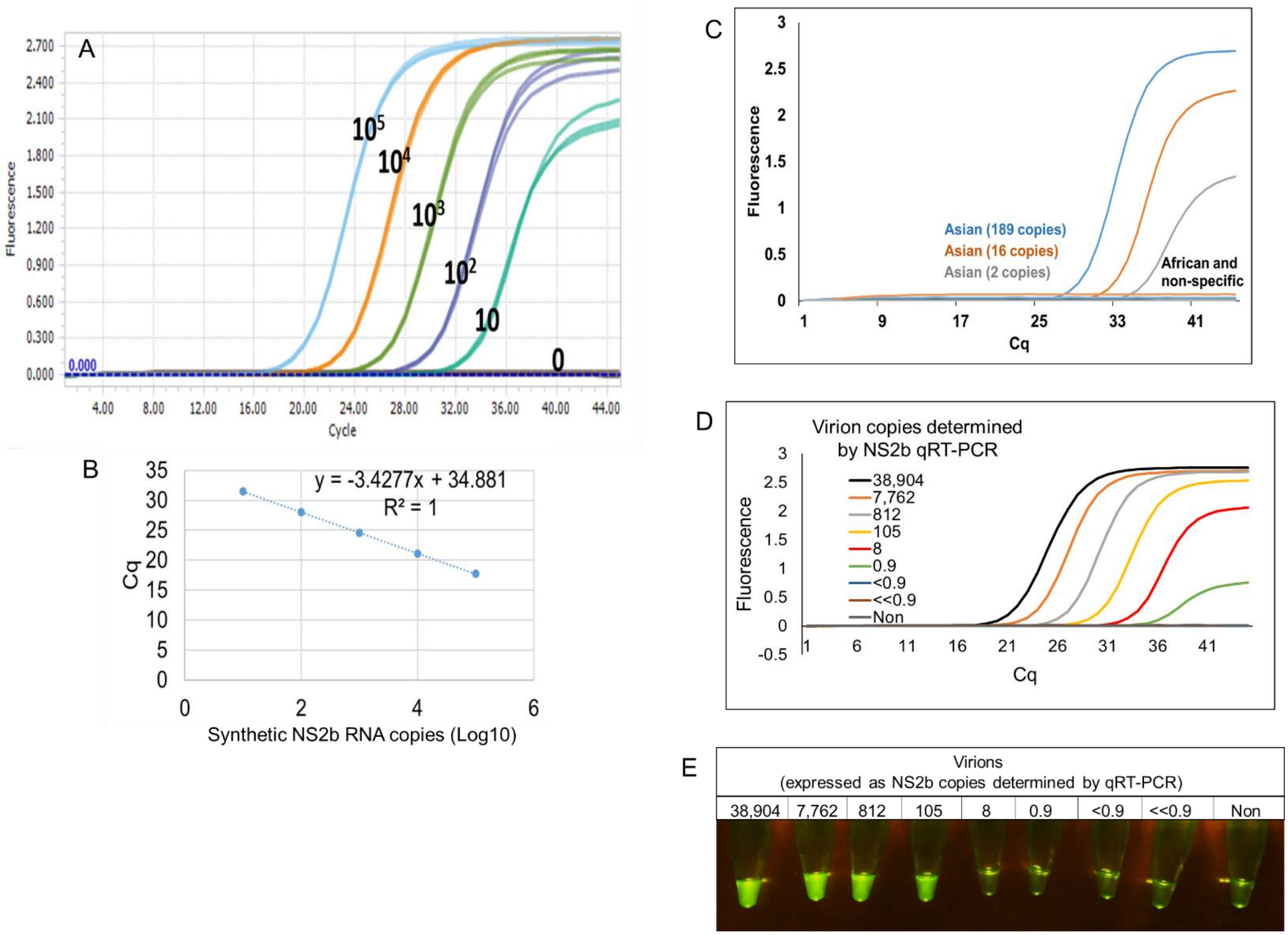

Supplementary Figure 2. Estimation of template copies in Asian lineage ZIKV genomic RNA and ZIKV virions. (A and B) Standard curve analysis of NS2b TaqMan qRT-PCR assay using indicated copies of synthetic NS2b RNA template. (C) NS2b TaqMan qRT-PCR analysis of an Asian lineage ZIKV genomic RNA. Real-time amplification curves for different genomic RNA copies are depicted as blue (189 copies), orange (16 copies), and gray (2 copies) traces. Lower template copies or non-specific templates were not detected. Genome copy numbers were estimated using a standard curve analysis (B) of different amounts of synthetic NS2b RNA. (D) NS2b TaqMan qRT-PCR analysis of Asian lineage ZIKV virions. Template copies were estimated using standard curves depicted in panel B. (E) Detection limit of degenerate multiplex LAMP-2GO assays for Asian lineage ZIKV virions. ZIKV virion copies (as deduced by NS2b TaqMan qRT-PCR in panel B) in each reaction are indicated. 'Non': non-specific virions; 10^6 $TCID_{50}$. Smartphone image was taken after 2 h of amplification.

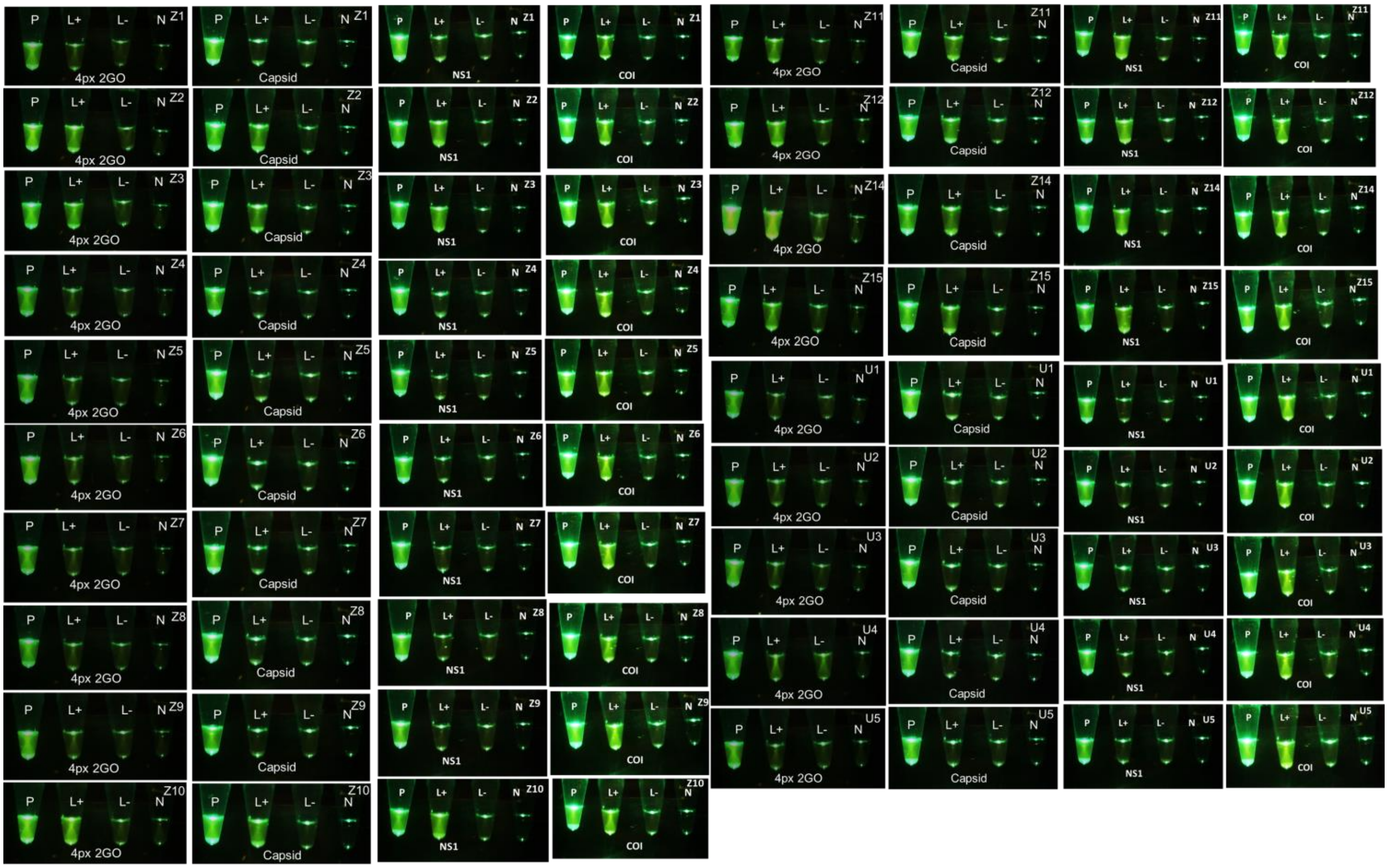

Supplementary Figure 3. Detection of Zika virus infected mosquitoes using individual and multiplex degenerate LAMP assays. Zika virus infected ('Z') and uninfected ('U') *Aedes aegypti* mosquitoes were directly analyzed using degenerate NS1 and capsid LAMP-OSD assays or with multiplex LAMP-2GO ('4px 2GO') assays. Assay type is indicated at the bottom of each individual panel. As a positive control mosquitoes were tested using the *Ae. aegypti coi* LAMP-OSD assay. Smartphone images acquired after 2h of amplification are depicted. P: positive control; L+: mosquito analyte with LAMP primers; L-: mosquito analyte without LAMP primers; N: no template control.
